# Could Aspirin Treatment Modify the Assessment of the Uterine Arteries?

**DOI:** 10.1055/s-0042-1742411

**Published:** 2022-02-09

**Authors:** Gabriela Marisol Vallejo, Montserrat Uriel, Alexandra Porras-Ramírez, Ximena Carolina Romero

**Affiliations:** 1 Universidad El Bosque, El Bosque Research Group of Maternal Fetal, Medicine and Gynecology, Ecodiagnóstico El Bosque SAS, Los Cobos Medical Center, Bogotá, Colombia; 2 Research Group Community Medicine and Collective Health, El Bosque University, Los Cobos Medical Center, Bogotá, Colombia

**Keywords:** aspirin, pre-eclampsia, ultrasonography, doppler, uterine artery, aspirina, pré-eclâmpsia, ultrassonografia, doppler, artéria uterina

## Abstract

**Objective** To analyze whether acetylsalicylic (ASA) intake modifies the mean uterine arteries pulsatility index (UtA-PI) at the 2 ^nd^ or 3 ^rd^ trimester in a cohort of pregnant women with abnormal mean UtA-PI at between 11 and 14 weeks of gestation.

**Methods** This is a retrospective cohort study. Singleton pregnancies with abnormal mean UtA-PI at between 11 and 14 weeks of gestation were studied. The participants were divided into 3 groups: 1) If the participant did not take ASA during pregnancy; 2) If the participant took ASA before 14 weeks of gestation; and 3) If the participant took ASA after 14 weeks of gestation. The mean UtA-PI was evaluated at the 2 ^nd^ and 3 ^rd^ trimesters, and it was considered to improve when it decreased below the 95 ^th^ percentile. The prevalence ratio (PR) and the number needed to treat (NNT) were calculated.

**Results** A total of 72 participants with a mean UtA-PI > 95 ^th^ percentile at the 1 ^st^ trimester of gestation were evaluated. Out of the 18 participants who took ASA, 8 participants started it before 14 weeks of gestation and 10 after. A total of 33.3% of these participants had improved the mean UtA-PI at the 2 ^nd^ and 3 ^rd^ trimesters of gestation, although it was not statistically significant ( *p* = 0.154). The prevalence ratio was 0.95 (95% confidence interval [CI]: 0.31–1.89), but between the 1 ^st^ and 2 ^nd^ trimesters of gestation, the PR was 0.92 (95%CI: 0.21–0.99) and it was statistically significant.

**Conclusion** The present work demonstrates a modification of the mean UtA-PI in participants who took ASA compared with those who did not. It is important to check if ASA can modify the normal limits of uterine arteries because this could have an impact on surveillance.

## Resumo

**Objetivo** Analisar se a ingestão de acetilsalicílico (ASA) modifica o índice médio de pulsatilidade das artérias uterinas (UtA-PI) no 2° ou 3° trimestre em uma coorte de gestantes com média anormal de UtA-PI entre 11 e 14 semanas.

**Métodos** Este é um estudo de coorte retrospectivo. Gravidezes únicas com média anormal de UtA-PI entre 11 e 14 semanas foram estudadas. As participantes foram divididas em 3 grupos: 1) Se a participante não tomou ASA durante a gravidez, 2) Se a participante tomou AAS antes das 14 semanas e 3) Se a participante tomou ASA após 14 semanas. A média do UtA-PI foi avaliada nos 2° e 3° trimestres e considerou-se que melhorava quando diminuía < 95° percentil. Foram calculados a razão de prevalência (RP) e o número necessário para tratar (NNT).

**Resultados** Foram avaliadas 72 participantes com média de UtA-PI > 95° percentil no 1° trimestre de gravidez. Das 18 participantes que tomaram ASA, 8 participantes começaram antes de 14 semanas e 10 depois. Um total de 33,3% desses participantes melhoraram a média de UtA-PI nos 2° e 3° trimestres, embora não tenha sido estatisticamente significante ( *p* = 0,154). A razão de prevalência foi de 0,95 (intervalo de confiança [IC95%]: 0,31–1,89), mas entre os 1° e o 2° trimestres, a RP foi de 0,92 (IC95%: 0,21–0,99) e foi estatisticamente significativa.

**Conclusão** O presente trabalho demonstra uma modificação da média de UtA-PI em participantes que faziam uso de ASA em comparação com aqueles que não faziam. É importante verificar se o ASA pode modificar os limites normais das artérias uterinas porque isso pode ter um impacto na vigilância.

## Introduction

Pre-eclampsia (PE) is an obstetric complication associated with remarkable maternal and perinatal morbidity and mortality worldwide. 1 2 About 18% of maternal deaths are secondary to hypertensive disorders of pregnancy, and in developing countries it can reach up to 35%. 3 4 5 6 7 The pathophysiology of PE is not yet fully understood, but it is considered that one of the most important factors is placental ischemia, secondary to an inadequate trophoblastic invasion of the maternal spiral arteries with subsequent vasoconstriction, release of antiangiogenic factors, and endothelial damage, which will be reflected in the clinical manifestations of the disease. 8

The measurement of the mean uterine arteries pulsatility index (UtA-PI) using the Doppler technique is an indirect validated indicator of trophoblastic invasion and placental perfusion and can be altered in the early stages of gestation. 9 10 11 12 Determination of the mean UtA-PI at the 1 ^st^ trimester of gestation is one of the elements of an algorithm that also combines maternal factors, mean arterial pressure (MAP), maternal serum pregnancy-associated plasma protein-A (PAPP-A) and placental growth factor (PlGF). This model detects ∼ 75% of the patients who will suffer preterm PE with a false positive rate of 10%. 13 14 15 Additionally, it described that the combined screening early in the 3 ^rd^ trimester (30 to 34 weeks) predicted almost all cases of preterm PE and half of term PE with a false positive rate of 5%. 16

Acetylsalicylic is an anti-inflammatory medication that could decrease resistance in the uteroplacental blood flow by inhibiting thromboxane-mediated vasoconstriction and permitting prostacyclin-mediated vasodilation. 17 18 Recently, The Combined Multimarker Screening and Randomized Patient Treatment with Aspirin for Evidence-Based Pre-eclampsia Prevention (ASPRE) trial reported that ASA at a dose of 150 mg per day, started between 11 and 14 weeks, reduces the incidence of preterm PE by 62% in patients with a high risk of developing PE, 19 but a meta-analysis aimed at assessing the impact of the start of ASA intake at the 2 ^nd^ trimester in women with abnormal UtA-PI showed no benefit regarding the reduction of PE, suggesting that its action is lost after week 16. 20 Currently, there are not enough data to explain how the mean UtA-PI behaves as the gestation progresses when women take ASA before 14 weeks and it has not been explored whether ASA could modify the 95 ^th^ percentile of mean UtA-PI when patients with high risk of PE at the 2 ^nd^ and 3 ^rd^ trimesters are followed-up. The present study aimed to analyze whether ASA intake could modify the mean UtA-PI at the 2 ^nd^ or 3 ^rd^ trimesters in a cohort of pregnant women with abnormal mean UtA-PI (PI > 95 ^th^ percentile) at the 1 ^st^ trimester.

## Methods

This was a retrospective cohort study of prospectively collected data from singleton pregnancies attending for a routine ultrasound scan at the 1 ^st^ , 2 ^nd^ , and 3 ^rd^ trimesters performed between 2014 and 2017 in an obstetric ultrasound center attached to El Bosque University. All participants with abnormal Doppler of uterine arteries, considered as mean UtA PI > 95 ^th^ percentile between 11 and 13 + 6 weeks (dated by CRL), were included. The exclusion criteria were 1) patients with losses in the follow-up of UtA Doppler at the 2 ^nd^ and 3 ^rd^ trimesters; 2) participants with multiple pregnancy; and 3) maternal age < 14 years old.

The following demographic characteristics were obtained: maternal age, ethnic origin, body mass index (BMI), comorbidities, smoking during pregnancy, primiparous, and personal and family history of preeclampsia. The visits were made between 11 and 13 + 6 weeks, between 18 and 23 + 6 weeks, and between 28 and 31 + 6 weeks.

**Fig. 1:**
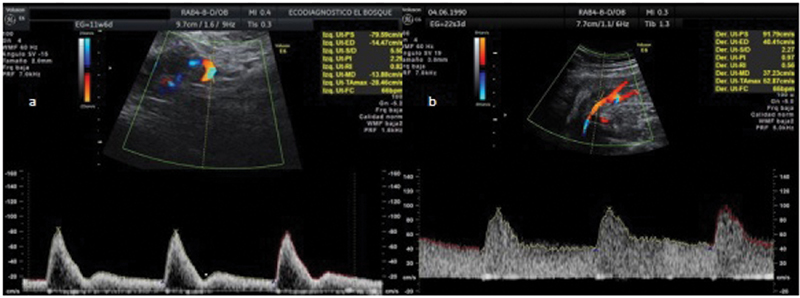
Uterine arteries pulsatility index measurements. Source: Sotiriadis et al., 23 Plasencia et al., 24 Martin et al., 25 and Gómez et al. 26

The measurement of the mean value of the left and right UtA-PI was performed by specialists in maternal fetal medicine certified by the Fetal Medicine Foundation, using color Doppler ultrasound according to a standardized protocol for each of the trimesters and following the international guidelines. 21 22 For the 1 ^st^ trimester examination, a midsagittal section of the uterus with a transvaginal transducer was obtained and the cervical canal and internal cervical OS were identified. The transducer was then moved laterally, and color flow mapping was used to identify each paracervical vascular plexus and the right and left UtA adjacent to the cervix at the level of the internal cervical OS. The samples of pulsed Doppler were established at 2 mm to cover the entire vessel with an insonation angle < 30°. When three similar consecutive waveforms were acquired, the PI was measured, and the mean PI of both arteries was calculated and registered ( Fig. 1 ). 23 24 25 26

**Fig. 2:**
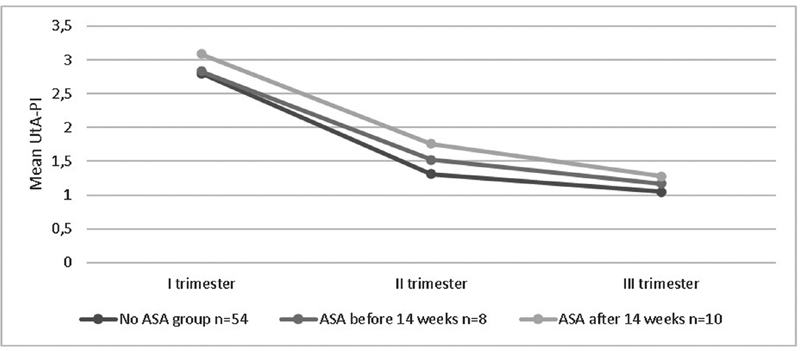
Mean UtA-PI of each trimester in the groups: non-ASA, ASA before week 14, and ASA after week 14. **Source:** Sotiriadis et al., 23 Plasencia et al., 24 Martin et al., 25 and Gómez et al. 26

Pregnancies at 18 to 23 + 6 weeks and between 28 and 31 + 6 weeks were examined transabdominally with the probe placed parallel to the iliac crest and color Doppler imaging was used to identify the UtA, which was measurement 1 cm distal from the crossover point of the iliac artery with the UtA. In all cases, with an angle < 30°, the pulsed Doppler gate was placed over the whole width of the vessel with a maximum size of 2 mm. Angle correction was then applied and the signal was updated until three similar consecutive waveforms had been acquired. The PI of the left and right arteries was measured, and the mean PI was calculated and registered ( Fig. 2 ). 23 24 25 26

Since the main prospective study was not an intervention study, the participants attended with their treating doctor, who defined the start of ASA at a dose of 100 mg orally (only dosage form available in Colombia at that time) and the gestational age of its onset. At each follow-up visit, patients were questioned regarding ASA intake and if the answer was affirmative, the gestational age of onset reported by the participant was recorded.

The participants were divided into three groups: 1) if the participant did not take ASA during pregnancy, 2) if the participant had started ASA intake before week 14, and 3) if the participant took ASA after week 14. Although some studies indicate that the optimal time for initiating ASA administration is ≤ 16 weeks, in the present study, it was established a cutoff point at 14 weeks based on the recently published ASPRE study. 19

The outcome was defined by the improvement of mean UtA-PI < 95 ^th^ percentile for the gestational age at the 2 ^nd^ and/or 3 ^rd^ trimesters using the ranges of reference informed by Gómez et al. 26 In the present study, the effectiveness of ASA was defined by achieving this outcome.

Comparisons of prevalence ratios (PRs) 27 28 29 30 were analyzed and, to measure the effectiveness of ASA intake, the number needed to treat (NNT) was used. IBM SPSS Statistics for Windows, Version 24 (IBM Corp., Armonk, N.Y., USA) was used for all data analysis.

The study was approved by the local ethics committee of the Universidad El Bosque (resolution number 006–2014) and by the ethics committee of the Kennedy Hospital Services Unit, and the participants signed the informed consent form. In the present study, the Principles of Helsinki and of resolution 8430 from October 4, 1993, were taken into account. 31 32

## Results

**Fig. 3:**
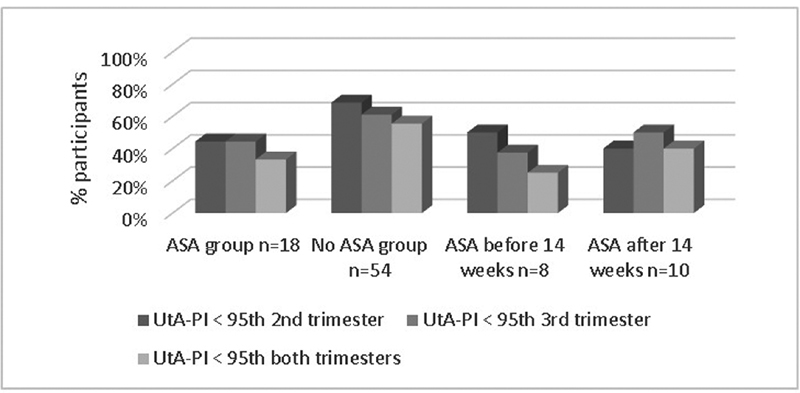
Performance of the mean UtA-PI in the three groups. **Source:** Sotiriadis et al., 23 Plasencia et al., 24 Martin et al., 25 and Gómez et al. 26

A total of 78 participants of the main prospective study had the mean UtA-PI > 95 ^th^ percentile, a total of 6 participants were excluded (5 participants who presented with late abortions and another who did not attend to the follow-up appointment). Among the 72 participants evaluated, it was found that 18 took ASA (25%), only 8 of whom started ASA intake before 14 weeks, and 10 after 14 weeks (broken down as follows: 2 participants started ASA at 16 weeks, 1 at week 18, 1 at week 20, 5 at 22 weeks, and 1 at 28 weeks). Table 1 compares the social and clinical characteristics of the women studied. Fig. 2 shows the mean UtA-PI of each trimester in each group. It was found that out of the total of women who used ASA, 33.3% ( *n* = 6) had an improvement of the mean UtA-PI at the 2 ^nd^ and 3 ^rd^ trimesters, even though it was not statistically significant ( *p* = 0.154); and from these 6 participants, 33.3% ( *n* = 2) started the intake of ASA before 14 weeks ( *p* = 0.236) ( Fig. 3 ). 23 24 25 26

**Table 1:** Social and clinical characteristics of the group of participants

Characteristics	No-ASA *n* = 54	ASA group before 14 weeks *n* = 8	ASA group after 14 weeks *n* = *10*
Maternal age (years old)	27.9 ± 7.3	31.2 ± 7.1	25.1 ± 5.6
First-trimester GA assessment (weeks)	12.8 ± 0.6	12.7 ± 5.8 **	12.4 ± 0.6
Second-trimester GA assessment (weeks)	21.7 ± 1.3	20.9 ± 0.8	21.3 ± 1.1
Third-trimester GA assessment (weeks)	29.5 ± 1.0	28.9 ± 0.6	28.9 ± 0.6
Ethnic origin			
Mixed	54 (100)	8 (100)	10 (100)
Primigravida	18 (33.3)	0 (0)	4 (40)
Comorbidities			
Diabetes mellitus	0 (0)	0 (0)	1 (10)
Chronic hypertension	1 (1.85) *	3 (37.5)	1 (10)
Obesity	4 (7.4)	2 (25)	0 (0)
Chronic renal failure	0 (0)	0 (0)	1 (10)
Anemia	1 (1.85)	1 (12.5)	0 (0)
Hypothyroidism	0 (0) *	0 (0)	4 (40)
Low maternal weight	4 (7.4)	1 (12.5)	1 (10)
Smoker			
Yes	9 (16.6)	2 (25)	3 (30)
No	45 (83.3) *	6 (75)	7 (70)
Personal history of PE	2 (3.7) *	3 (37.5)	2 (20)
Family history of PE	7 (12.9) *	3 (37.5)	4 (40)
BMI (kg/m ^2^ )	24.9 ± 4.1	27.5 ± 5.8	23.8 ± 3.2

Abbreviations: APS, antiphospholipid syndrome; ASA, acetylsalicylic acid; BMI, body mass index; GA, gestational age; PE, preeclampsia; SLE, systemic erythematous lupus.

Data are prasented as mean ± standard deviation or as *n* (%).

* Indicates statistically significant differences between aspirin versus no aspirin groups

** Indicates statistically significant differences between groups aspirin before 14 weeks and after 14 weeks.

The PR that was found for the effectiveness of ASA at the 2 ^nd^ and/or 3 ^rd^ trimesters was 0.95 (95% confidence interval [CI]: 0.31–1.89). On the other hand, the PR found for the effectiveness of ASA in the improvement of mean UtA-PI at the 1 ^st^ trimester compared with the 2 ^nd^ trimester was 0.92 (95%CI: 0.21–0.99) which was was statistically significant. In the ASA before the 14 ^th^ week group, 50% of the patients had improved the mean UtA-PI at the 2 ^nd^ trimester. The NNT was 24, that is mean that, out of every 24 patients, 1 patient improves the mean UtA-PI at the 2nd trimester in the aspirin group. The PR found for the effectiveness of ASA for the improvement the mean UtA-PI when it was abnormal at the 1 ^st^ and 2 ^nd^ trimesters and then normal at the 3 ^rd^ trimester, was 0.96 (95%CI: 0.19–0.98), which was also statistically significant. To achieve decrease and normalization of the mean UtA-PI at the 1 ^st^ trimester compared with the third trimester, it was found that PR was 0.67 (CI 95% 0.38–1.16), although this difference was not statistically significant. In the ASA before the 14 ^th^ week group, 37.5% of the patients had improvement in the mean UtA-PI at the 3 ^rd^ trimester. The NNT was 22. In addition, it was found that women who did not take ASA had a PR of 1.62 (95%CI: 0.87–3.01), continuing with the abnormal mean UtA-PI at the 3 ^rd^ trimester, although this difference was not statistically significant; and women who took ASA after 14 weeks had a PR of 1.14 (95%CI: 0.46–2.78), continuing with the mean UtA-PI > 95 ^th^ percentile at the 3 ^rd^ trimester, although this difference was not statistically significant either ( Table 2 ).

**Table 2:** Prevalence ratio according to each group

Characteristics	Prevalence ratio	95%CI
When the mean UtA PI was abnormal at the first trimester and then it was normal at the second and/or third trimesters	0.95	0.31–1.89
When the mean UtA PI was abnormal at the first trimester and then it was normal at the second trimester	0.92	0.21–0.99
When the mean UtA PI was abnormal at the first and second trimesters and then it was normal at the third trimester	0.96	0.19–0.98
When the mean UtA PI was abnormal at the first trimester and then it was normal at the third trimester	0.67	0.38–1.16
No ASA intake or started it after 14 weeks of gestation	1.62	0.87–3.01

Abbreviations: ASA, acetylsalicylic acid; CI, confidence interval; UtA PI, uterine arteries pulsatility index.

## Discussion

Worldwide, PE complicates 2 to 3% of all pregnancies; but in developing countries, the incidence increases up to 8%, being the main cause of maternal death in Colombia in 2017. 1 3 5 6 7 Although the pathophysiology of PE has not been completely understood, there is evidence that impaired trophoblastic invasion of the spiral arteries is one of the underlying causes. Abnormal UtA Doppler at the 1 ^st^ trimester is a reflection of increased resistance to flow and a nonspecific biomarker of prediction of early PE. 25 The onset of ASA at the 1 ^st^ trimester of pregnancy has shown to have an effect on the reduction of the incidence of preterm PE in high-risk patients 19 and of the incidence for small for gestational age (SGA) and fetal growth restriction (FGR). 20 However, there are very few studies that evaluate the performance of the mean UtA-PI in patients taking ASA.

In the present study, there were statistically significant changes in the assessment of the mean UtA-PI of participants taking ASA compared with those who did not take it. This is reflected in the participants with abnormal mean UtA-PI at the 1 ^st^ trimester who had 0.92 times more probability of normalizing the mean UtA-PI at the 2 ^nd^ trimester compared with those who did not take ASA as a factor protector. Additionally, regarding improvement of the mean UtA-PI at the 3 ^rd^ trimester, it was found that women who took ASA had 0.67 times more probability of decreasing the mean UtA-PI at the 3 ^rd^ trimester, although this difference was not statistically significant. In this case, for every 22 participants treated with ASA, an improvement of the mean UtA-PI was obtained in 1 woman between the 1 ^st^ and 3 ^rd^ trimesters.

Furthermore, it is important to take into account what happened with the uterine arteries of the participants who did not take ASA or did so after 14 weeks, who were, respectively, 1.62 and 1.14 times more likely of continuing with the mean UtA PI > 95 ^th^ percentile at the 3 ^rd^ trimester, as a risk factor, although this difference was not statistically significant.

The results of the present study differ with those of the study by Scazzocchio et al, 9 a randomized trial that included 155 patients with abnormal 1 ^st^ trimester UtA Doppler, in which 75 patients received placebo and 80 received ASA. They evaluated whether low doses of ASA administered from the 1 ^st^ trimester improved trophoblastic invasion assessed by UtA Doppler at 28 weeks, and no significant effect was found. However, these authors compared the mean UtA-PI between the two groups to determine if there were significant differences and not the normalization of Doppler, as in our study. 9 On the other hand, in the study of Haapsamo et al. 33 that included 37 patients who had undergone 2 techniques of assisted reproduction (in-vitro fertilization and intracytoplasmic sperm injection), it was observed that the PI of the right and left UtAs at 18 weeks was lower in the ASA group compared with the placebo group when ASA was started before pregnancy ( *p* < 0.05), but normalization of the UtA-PI was not reported. Therefore, the results of the aforementioned studies cannot be compared with those of our study because they did not evaluate the improvement in UtA-PI and it may be worth considering that if this parameter changes, it also changes the normal limits in patients who take ASA from the 1 ^st^ trimester.

In the present study, the outcome of presentation of diseases related to placentation such as hypertensive disorders of pregnancy or fetal growth disturbances were not evaluated, so it cannot be said that the normalization of the mean UtA-PI in these participants was associated with a decrease in the onset of these disorders. There could be a selection bias since the data in this study are extracts from the main study whose objective was to evaluate PE screening and correspond to a specific population. Furthermore, random error due to the small sample size of this study is acknowledged.

More studies with a greater number of participants would be needed to determine if the change of the mean of UtA-PI as the pregnancy progresses is clinically relevant in the assessment of patients with risk of PE or of intrauterine growth restriction, and if it is justified to change the reference values of the 95 ^th^ percentile, discriminating whether the participants were taking ASA or not.

## Conclusion

The intake of ASA in patients with abnormal UtA-PI at the 1 ^st^ trimester was associated with a normalization statistically significant of this parameter at the 2 ^nd^ or 3 ^rd^ trimesters. More studies are needed to determine if these results could modify the usual clinical obstetrical care.
